# Development and evaluation of BDNF-loaded PCL/PVA two-layer nerve guidance conduit with enhanced biomechanical and biological properties for peripheral nerve regeneration

**DOI:** 10.1016/j.heliyon.2025.e42792

**Published:** 2025-02-19

**Authors:** Massoumeh Jabbari Fakhr, Mohsen Eslami Farsani, Leyla Fath-Bayati, Reihaneh Seyedebrahimi, Mehdi Sadrjahani, Fatemeh Kavakebian, Alireza Rezapour

**Affiliations:** a Department of Tissue Engineering and Applied Cell Sciences, School of Medicine, Qom University of Medical Sciences, Qom, Iran; b Department of Anatomy, School of Medicine, Qom University of Medical Sciences, Qom, Iran; c Faculty of Textile Engineering, Urmia University of Technology, Urmia, Iran; d Cellular and Molecular Research Centre, Qom University of Medical Sciences, Qom, Iran

**Keywords:** BDNF, Two-layer nerve guidance conduit, 3D model, Nanofiber, Peripheral nerve regeneration

## Abstract

The repair of damaged peripheral nerves and the following restoration of functionality remain significant therapeutic challenges. Hollow nerve conduits currently available do not align with the ideal human model. Successfully mending nerve gaps requires incorporating biomimetic and functional features into neural conduit design. In this research, a new two-layer conduit that combines topographic support and controlled growth factor release was developed. We used a two-layered framework to amplify the mechanical reinforcement and reduce the risk of tissue collapse post-grafting. The hollow nerve conduits were fabricated through three-dimensional printing, employing Polycaprolactone (PCL) and a slowly biodegradable nanofiber for the intraluminal brain-derived neurotrophic factors (BDNF)-loaded polyvinyl alcohol (PVA)/PCL core-shell. The contact angle was indicated to show the hydrophilicity properties and degradation rate for biocompatibility. The scanning electron microscope **(**SEM) images were analyzed to determine the fiber's diameters, structure morphology, and stem cell adhesion. The performance of core-shell conduits was investigated in human dental pulp stem cells **(**hDPSC) culture and their differentiation into Schwann cells (SCs) *invitro*. The vitality of samples was assessed using SEM, MTT assay, and differentiation potential with real-time and Immunofluorescence staining techniques. *Invitro* cumulated BDNF release followed the Korsmeyer-Peppas model, demonstrating a strong correlation coefficient of 0.981. Real-time analysis showed that after 14 days of induction, the expression of S100 increased 5.89-fold. We concluded that core-shell PCL/PVA nerve guidance conduits can encourage the adhesion and proliferation of hDPSCs and create the ideal environment for increasing cell survival. Also, the sustained release of BDNF within conduit walls promoted differentiation toward SC.

## Highlights

• hDPSCs show mesenchymal stem cell markers CD 73 and CD 90 in >99 % of isolated cells.
• The core-shell nanofiber design supports sustained BDNF release over 14 days.
• Optimized NGC outer layer porosity enhances surgical suturing and tissue growth.
• Immunofluorescence confirms Schwann cell differentiation of hDPSCs on the conduit.
• Two-layer NGC enables longitudinal cell growth, aiding Schwann-like cell development.

## Graphical abstract

**Figure:**
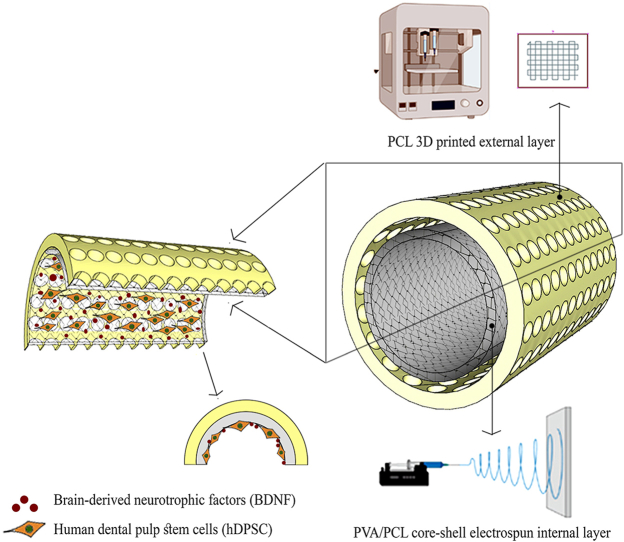

## 1 Introduction

Peripheral nerve injury (PNI) is a prevalent disorder of the peripheral nervous system that can significantly impact a patient's quality of life. Despite the considerable advancements in treating peripheral nerve defects, auto-graft transplantation remains the standard treatment method. It is pertinent to point out that this particular therapeutic approach entails some limitations that warrant careful consideration. One such limitation is the requirement for a surgical procedure to remove a nerve, which, in turn, may be associated with complications such as infection, neuroma formation, and scarcity of donor tissue. Giving due regard to these factors is crucial to ensure the procedure is effective [1,2].

Tissue engineering has emerged as a promising approach for managing peripheral nerve injuries. Nerve conduits have the potential to achieve a comparable level of regeneration performance to that of an autograft by connecting the proximal and distal nerve stumps [3,4]. Despite several generations of commercial nerve conduits developed for treating PNI, none contain internal conduit guidance structures for peripheral nerve regeneration [5]. Therefore, designing a suitable conduit is crucial in facilitating the progression of nerve regeneration. Previous studies have demonstrated that nerve guidance conduits (NGCs) possessing appropriate biochemical, physical, geometric, and chemotactic cues, as well as supporting cells and growth stimulants within their lumen, can mimic the properties of an autograft [[5], [6], [7], [8]].

An NGC can be designed as a complex three-dimensional structure comprising suitable biomaterials, cells, and biological factors that are utilized to replicate the natural environment of peripheral nerves and promote their regenerative processes. These structures are composed of numerous layers, facilitating a transfer from the conduit to the surrounding tissue and helping to reduce inflammation and promote the integration of the conduit with the adjacent tissue [9].

Cells are utilized in conduits due to their capability to secrete a consistent flow of neurotrophic factors and matrix proteins that aid in the regeneration of nerves [10]. Schwann cells (SCs) are one of the principal cells involved in repairing nerve tissue, forming the myelin of the peripheral nervous system. Studies show that SCs have biological activity and can produce nerve growth factors, secrete extracellular matrix, and guide axon growth [11]. Obtaining enough SCs for transplantation can be challenging due to their limited accessibility, slow growth in *ex vivo* conditions, and the adverse side effects of nerve harvesting [12]. These limitations have led researchers to explore alternative cell sources for repairing peripheral nerve lesions, including the use of embryonic stem cells (ESCs) [13], adipose-derived stem cells (ASCs) [14,15], bone marrow stem cells (BMCs) [16], and stem cells isolated from dental pulp (DPSCs) [17] all of which have shown promising results for nerve repair in various studies.

Polycaprolactone (PCL) and polyvinyl alcohol (PVA) are commonly used in peripheral nerve tissue engineering due to their non-toxic nature, biocompatibility, mechanical stability, manipulative flexibility, relatively affordable cost, and approval by the Food and Drug Administration (FDA) [3,18]. However, it is essential to address the main concerns regarding their weaknesses in biodegradability and cell binding motifs to achieve successful application in the fabrication process [5]. The combination of PCL and PVA within the conduit provides a supportive structure for the proliferation of neuronal cells. PVA, frequently employed as a water-soluble polymer in drug delivery systems, can also enhance the hydrophilicity of the structure's surface. Meanwhile, PCL can also provide strength and integrity to the structure. This approach can improve the final conduit's biological interaction and modify undesirable characteristics [5,19].

There are different techniques for constructing conduits. One of the most promising methods for peripheral nerve applications is using NGCs made from various electrospun biopolymers. These NGCs offer exceptional potential for peripheral nerve applications because of their large surface area to volume ratio, high porosity, density, and alignment of fibers, as well as their superior mechanical properties [5,20]. Due to the presence of electrospun fibers, this variant of NGC exhibits the capability of effective guidance along the longitudinal axis. However, it is unreliable in surgical procedures [1,21]. To fixate an NGC into the nerve stump, repetitively passing a suture through the wall of the NGC is necessary. Repeated penetrating makes the wall of the NGC disposed to destroy along the fiber orientation. Consequently, it is essential to progress NGCs with more tear-resistant walls to supply the surgery requirements [4].

In the present study, we developed and evaluated a new complex tubular structure that comprises two external and internal layer conduits. A three-dimensional (3D) porous outer wall of PCL is designed to provide mechanical strength and withstand surgical procedures, making it easier to implant. Also, PVA nanofibers on the inner layer are embedded to guide axonal extension and topographical and physical cues for neural differentiation and proliferation of neural cells [1 (Deng et al., 2022),4].

This study aimed to develop a nerve guidance conduit that would promote the regeneration of axons, modulate inflammation, and encourage SC differentiation. This conduit was designed to have the appropriate mechanical and biological properties for promoting nerve regeneration. The project was achieved by creating a two-layer PCL/PVA conduit containing DPSCs, continuously releasing brain-derived neurotrophic factors (BDNF) on the bioactive internal wall.

## 2 Material and method

### 2.1 The isolation and culture of the human dental pulp stem cells (hDPSCs)

The extracted third molars were obtained from patients who provided their consent. Then, they were placed into a solution of phosphate buffer saline (PBS) containing 3 % penicillin (100 U/ml) and streptomycin (100 mg/ml) (Sigma). This study was approved by the medical ethical committee of Qom University of Medical Science (IR.MUQ.REC.1400.180). All procedures were conducted using aseptic techniques under a biohazard laminar flow hood.

The teeth were cleaned with chlorhexidine and then fractured to collect pulp tissue. The pulp tissue was fragmented into smaller pieces with a scalpel and then subjected to enzymatic hydrolysis using a collagenase I (Sigma Aldrich SCR 103) solution (3 mg/ml in PBS) for 45 min in a shaker incubator. The resultant mixture was then centrifuged at 400 g for 10 min, and the cell pellets were collected and resuspended in DMEM (Bioidea), supplemented with 20 % fetal bovine serum (FBS) (Gibco), 100 U/ml penicillin, and 100 μg/ml streptomycin. The cells were cultured at 37 °C in 5 % CO_2_ and 95 % humidity. Once they reached 80 % confluency, primary DPSCs were subcultured using a 0.025 % trypsin/EDTA (Bioidea) solution [22].

### 2.2 Characterization of isolated hDPSCs

#### 2.2.1 Immunophenotypic analysis of hDPSCs

All hDPSCs were routinely tested for surface cell markers representing endothelial, hematopoietic, and mesenchymal stem cells. These markers included CD34, CD45, CD73, and CD90 (BD Biosciences). For analysis, cells from the third passage were collected, purified, and incubated with the specific labeled antibodies for 30 min at 4 °C. The data were then collected using a fluorescence-activated cell sorting (FACS) Canto II Flow cytometer (BD Biosciences) and evaluated using Flowjo_V10_CL analysis software.

### 2.3 Differentiation potential of DPSC *invitro*

#### 2.3.1 *Invitro* differentiation into osteocyte and adipocytes

5 × 10^4^ DPSCs were seeded in 12 wells in triplicate. After 24 h, the culture media were removed and replaced with adipogenic and osteogenic inductive media (Bioidea). The medium was changed twice weekly for 14 and 21 days, respectively. After differentiation, the medium was removed, and calcium mineral deposits were visualized using Alizarin Red. The adipogenic culture was stained with an oil-red O staining solution.

### 2.4 Conduit fabrication

#### 2.4.1 3D printed layer for external guidance paths of the nerve conduit

The external layer of conduits was fabricated using an extrusion-based 3D printer (Omid afarinan-PioneerX4). The structures were designed with computer-aided design (CAD) software (AutoCAD 2022), and the designs were exported as STL files to the Repetier-Host software V2.1.3. The cartridge was filled with PCL pellets (Sigma 440744), and the printing parameters were set as follows: a temperature of 80 °C, a pressure of 0.4 MPa, and a head movement speed of 0.4 mm/s. The printed structure matched the specified design, including the following characteristics: 20 mm, a length and width of 20 mm, a height of 1.06 mm, and an angle between layers of 90°. Specifically, three layers of a cuboid model were successfully printed.

#### 2.4.2 Electrospinning of BDNF-loaded PVA/PCL nanofibers for the inner guidance paths of the nerve conduit

**Fig. 1:**
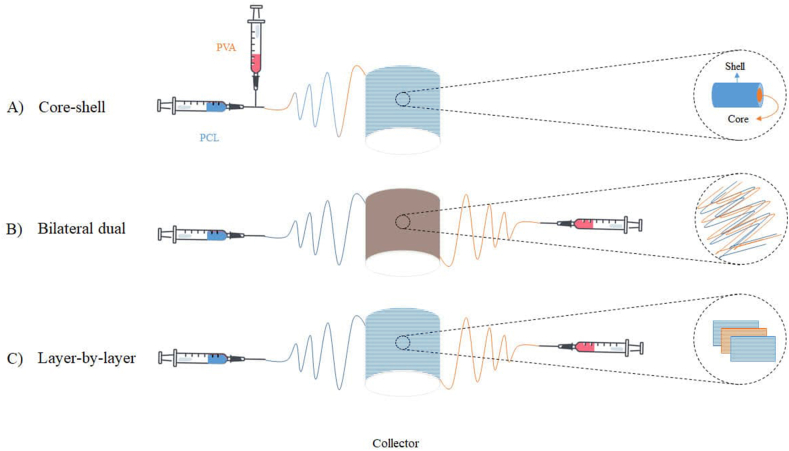
Schematic view of core-shell (A), bilateral dual (B), and layer-by-layer (C) electrospinning process.

The concentration of BDNF was added at 50 ng/ml in an aqueous solution containing 10 % PVA (w/v, Sigma Aldrich 363146). A solution consisting of PVA hydrogel and PCL (13 % w/v) Soluble in acetic acid and formic acid in a ratio of 80:20 (v/v) was loaded into separate syringes and continuously pumped through a metal blunt needle syringe. This process used three programs: core-shell, bilateral dual, and layer-by-layer (Fig. 1). The solution was fed through at a rate of 0.3–0.4 ml/h, with a distance of 15.0 cm between the tips and the collector. The Jet formation was achieved by applying a 13–15 kV voltage to a high-voltage power supply. Finally, the electrospun nanofibers were collected onto a 3D-printed layer attached to a rotating drum collector. The collector was operating at a speed of 1200 rpm. The successful integration of the inner and outer layers of the conduit was achieved through this process. The electrospinning process resulted in uniform fiber diameters and membranes with a thickness of 60 μm. The PCL/PVA structure was rolled to create a cylindrical conduit. The two ends of the PCL layer were joined meticulously together using a thermal method. The resulting conduit had an inner diameter of 3 mm, a length of 20 mm, and a wall thickness of 1.12 mm.

#### 2.4.3 Sterilization

Before cell seeding, the samples underwent sterilization through exposure to ultraviolet (UV) radiation within a chamber for 1 h per side. Subsequently, the samples were washed with PBS.

### 2.5 Characterization of NGC

#### 2.5.1 *Invitro* BDNF release study and kinetic modeling

The quantification of BDNF release from NGC was carried out using the Bradford assay over 14 days. NGC was incubated in a shaker incubator at 37 °C and immersed in PBS (pH 7.4). The medium on the samples was collected and replaced with fresh PBS at specific intervals of 3, 6, 12, 24, 48, 72, 168 h (7 days), and 14 days and stored at −20 °C for subsequent quantification.

The amount of released drug was then determined by Bradford kit and calculated as cumulative percent release. Sink conditions were maintained for the release study, and the experiments were performed in triplicate.

Additionally, to understand the *invitro* release mechanism, the release data were fitted to the Korsmeyer–Peppas model (Mt/M∞ = Kt^n^), where Mt is the amount of drug released at time t, M∞ is the total amount of drug released at infinite time, K is the rate constant and the exponent “n" represents the drug transport mechanism and can be used to evaluate the mechanism of diffusion.

#### 2.5.2 Scanning electron microscope (SEM) imaging

The morphology and thickness of the PVA layer, PCL sheet, and NGC, as well as the diameter and pore size, were observed by scanning electron microscopy. For SEM analysis, gold-coated samples were imaged using an XL-20 scanning electron microscope operating at an accelerating voltage of 7 kV.

#### 2.5.3 Attenuated total reflectance-Fourier transform infrared (ATR-FTIR) analysis

The PVA, PCL, and NGC were analyzed using a Nicolet Nexus 470 Attenuated Total Reflectance-Fourier Transform Infrared (ATR-FTIR). The ATR-FTIR spectrum was collected in the 4000–400 cm^−1^ range with 4 cm^2^ resolution averaging 120 scans.

#### 2.5.4 Tensile test

The mechanical properties of a scaffold can significantly impact both the surgical procedure and the success of the regenerative process. The tensile strength test was analyzed using a tensile test machine (STM-20) with a crosshead speed of 13 mm/min and a gauge length of 15 mm at room temperature (RT). For reproducibility of the data, three identical samples per group were measured.

#### 2.5.5 Wettability assessment

The surface hydrophilicity of the NGC at RT was assessed using an optical static contact angle analyzer (Jikan CAG-20 SE). The 3 μL water was dropped onto both internal and external layers, and the reaction was recorded by taking the image to investigate the water-in-air contact angle (n = 3).

#### 2.5.6 *Invitro* degradation rate

To measure hydrolytic degradation (according to ASTM F1635), samples of NGC were weighed (Wi) carefully with a high-resolution micro-balance (±0.0001 mg). Then, the samples were immersed in 0.01 M PBS solution (30 ml, pH = 7.4) and placed at 37 °C. Three samples were used for each time point of 1, 7, 14, 21, and 28 days after the samples dried at 40 °C. The final weight of samples (Wf) was measured carefully. The weight loss percentage was calculated using the formula: (Wf − Wi)/Wi × 100.

### 2.6 Cell culture in nerve guides conduits

#### 2.6.1 Cell seeding

The structures at the size of 20 × 20 mm were used for *invitro* studies. Each sample in the 6-well plates was seeded with a 3 × 10^5^ hDPSCs density. The samples loaded with the cells were then incubated in a growth medium.

#### 2.6.2 Cell attachment and morphology

The SEM images determined the morphology and cell attachment of samples. For this purpose, after 48 h, the cell culture medium was removed entirely, and the fixation was carried out by adding glutaraldehyde 2.5 % (v/v). After 2 h, the fixation solution was removed, and the cells were rinsed with deionized water. Graded concentrations of ethanol (60 %, 70 %, 80 %, and 90 %) were added for 10 min each, followed by 100 % ethanol for 30 min. The samples were then dried in a vacuum oven for 30 min, coated with gold, and finally, imaged.

#### 2.6.3 Cell viability assays

The non-cytotoxicity and biocompatibility of NGC were evaluated using the indirect MTT assay (3-(4,5-dimethylthiazol-2-yl)-2,5-diphenyltetrazolium bromide), in compliance with ISO 10993-5. The assessment was conducted at 1, 3, 5, 7, and 14 days. NGCs were immersed in complete media to facilitate the leaching process from the samples. The study evaluated the metabolic activity of fibroblast cells cultured in the presence of leachates extracted from samples over different times.

1 × 10^4^ L929 fibroblast cells were seeded and incubated at 37 °C and 5 % CO_2_ for 24 h. After this, the cells were treated with NGC extracts. 0.5 mg/ml of MTT solution was added and incubated for 4 h. The optical density was finally measured using a microculture plate reader (BioTek Instruments, America) at 570 nm.

The relative cell viability was calculated using the following equation:

$$\text{Cell}\;\text{viability}\;\%=\frac{{\text{OD}}_{\text{sample}}}{{\text{OD}}_{\text{negative}\;\text{control}}}$$

OD designates the optical density. A culture medium, including cells but no leachates, was tested as a negative control. Each measurement was repeated at least 3 times.

#### 2.6.4 Characterization of differentiated cells on NGCs

SEM imaging The cells are fixed accurately on the 14th day after differentiation to obtain precise SEM images of differentiated cells on NGCs.

### 2.7 Gene expression

#### 2.7.1 RNA isolation and cDNA synthesis

On day 14, total RNA was extracted from all study groups using SinaPure RNA-Sinaclon per the manufacturer's instructions. The extracted RNA concentration was detected for its quantity and quality by measuring the maximum absorbance at 260 nm with a NanoDrop (Thermo Fisher) and gel electrophoresis. Finally, the extracted total RNA was converted to complementary DNA (cDNA) using the Easy cDNA synthesis kit- Parstous.

#### 2.7.2 Quantitative real-time PCR assay

Quantitative assessments were conducted via Q-PCR on day 14 to measure the expression of Schwann cell-specific markers, S100, MBP, and GAPDH as the reference gene. The primers (Pishgam) were designed in NCBI, blasted, and listed in Table 1.

**Table 1:** The Real-time PCR primer sequences were used to screen Schwann cell genes.

GENE	FORWARD PRIMER	REVERSE PRIMER	AMPLICON SIZE
**S100**	TGGCCCTCATCGACGTTTTC	CAGTGTTTCCATGACTTTGTCCA	153
**MBP**	GTCCCTGAGCAGATTTAGCTG	GAATCCCTTGTGAGCCGATTT	104
**GAPDH**	ACAACTTTGGTATCGTGGAAGG	GCCATCACGCCACAGTTTC	101

The SYBER Green Master mix- VERNER Kit was utilized to perform Q-PCR on study groups and analyze the expression of SC-specific markers: S100 and MBP. Each reaction contained 20 μl (total reaction) of mixture solution, including 12.5 μl of Q-PCR 2 × Master Mix with syber green, 5 pmol concentration of each primer, 4.5 μl deionized water, and 2 μl of cDNA. The time and temperature settings were programmed as follows: 95 °C for 15 min as the holding time, 30 s of denaturation at 95 °C, 30 s at 54–57 °C to anneal the primers, 20 s of extension at 72 °C, and a gradient temperature of 60–95 °C was used for melting of productions with a ramping rate of 0.3 °C.

Reactions were amplified with 40 cycles in Rotorgen 6000 QIAGEN (Germany). All reactions were repeated three times in the experiment. Finally, the relative expression result was analyzed using the Pfaffi method in real-time PCR using MyGo Pro PCR software 3.4.

#### 2.7.3 Immunofluorescence

IF was used to confirm that hDPSCs differentiate into Schwann cells on NGC based on the presence of SC-specific protein S100 and MPB. On day 14, the differentiated hDPSCs on substrates were fixed with 15 % formaldehyde at RT, followed by the preparation of paraffin-embedded blocks. Three-micron sections were cut and placed on coated glass slides, subsequently dewaxed utilizing xylene, subjected to a rinse in a graded series of alcohol, and rehydrated in distilled water; antigen retrieval (AR) was conducted employing a citrated buffer.

Cells were incubated with the following primary antibodies: Anti S100 (QR031) and anti-MB (Sigma M9434) at RT for 2 h. Subsequently, the cells underwent a 1 h incubation at RT with FITC-conjugated secondary immunoglobulin G (anti-mouse, AB1015, or anti-rat, A11006). Cell nuclei were labeled with DAPI (Thermo Fischer) added to the secondary antibody solution at a ﬁnal concentration of 8 μM for 5 min at RT. Samples were examined under a ﬂuorescence microscope.

## 3 Result and discussion

### 3.1 Characterization of isolated hDPSCs

**Fig. 2:**
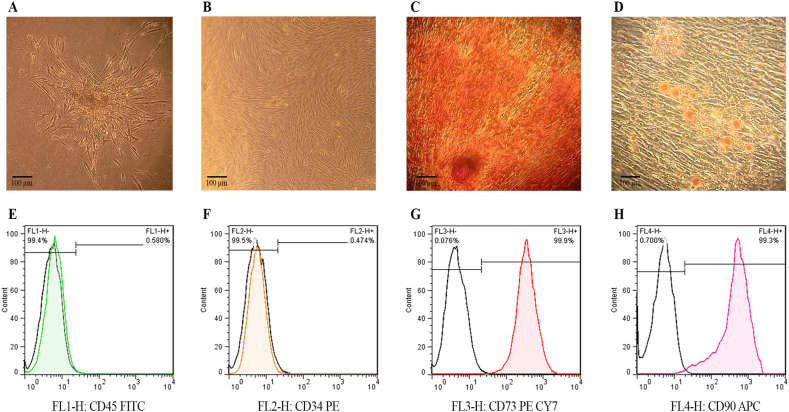
Characterization of isolated hDPSCs: fibroblast-like morphology of hDPSCs after one week (A) and two weeks of isolation (B), Alizarin Red staining (C), Oil red staining (D), flow cytometry analysis (E–H).

The hDPSCs were characterized using light microscopy based on their fibroblast-like morphology. After three passages, the DPSCs formed a confluent fibroblast-like monolayer (Fig. 2A and B). The phenotypic characteristics were determined by observing calcium deposits using Alizarin Red staining (Fig. 2C). In addition, lipid-laden vacuoles were confirmed using oil red O, demonstrating osteogenic and adipogenic features, respectively (Fig. 2 D).

FACS analysis demonstrated a negative response for hematopoietic and epithelial stem cell markers CD 45 and CD 34 (Fig. 2E and F). On the other hand, the results revealed that a significant proportion of the cells strongly express the mesenchymal stem cell markers CD 73 and CD 90 (Fig. 2G and H). According to this result, DPSCs were identified in more than 99 % of the isolated cell population.

### 3.2 Characterization of NGC

**Fig. 3:**
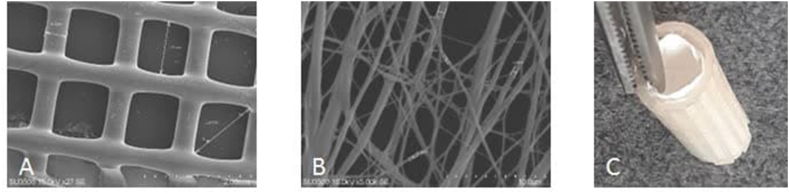
SEM image of conduit: morphology of 3D printed structure (A), electrospun fibrous mat in internal layer (B), and macroscopic view of two-layer NGC (C).

The final physical and chemical properties of developed NGCs are significantly influenced by their morphology, primarily shaped by 3D and electrospinning parameters. Upon SEM analysis, it was observed that the internal layer consists of highly porous and uniform nanoscale fibrous mats. Conversely, the outer layer exhibits macro pores and specific dimensions, including an average layer thickness of 600 μm and a fiber diameter of about 400 μm (Fig. 3. A). Furthermore, the inner layer features fibers with an average diameter of 444 nm (Fig. 3. B).

The peripheral nervous tissue is longitudinally arranged, and multiple studies have highlighted the importance of topographic and aligned structures in facilitating axonal growth within nerve conduits [23]. The porosity must align with host tissue properties to ensure mechanical stability for tissue growth. Therefore, optimal pore sizes vary based on tissue type [24]. The 3D optimal open porosity of the NGC's outer layer facilitates surgical suturing and enhances nutrient transfer and vascularization for neo-tissue formation [25].

### 3.3 *InVitro* drug release and kinetic modeling

**Fig. 4:**
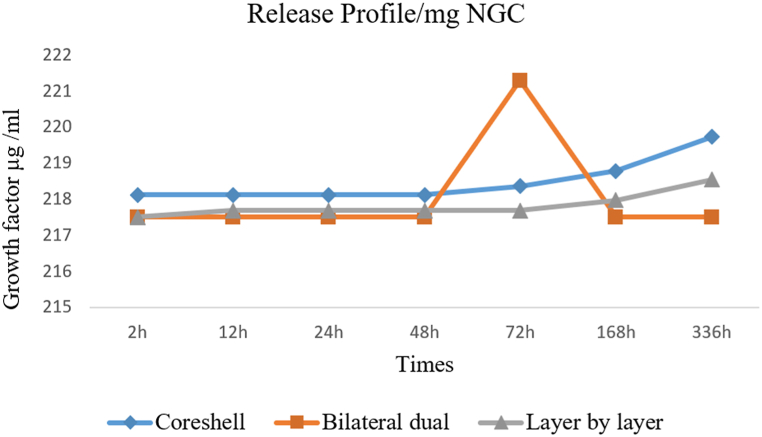
Sustained release of growth factor delivery system in 3 frameworks of electrospinning.

The pattern of nanofiber synthesis significantly influences the release rate of growth factor. Hence, this study utilized PVA nanofibers loaded with BDNF to establish optimal release profiles for creating internal conduction pathways within the neural tube. The process was achieved by implementing core-shell, layer-by-layer, and double-layer structures. *Invitro* studies revealed distinct variations in the release of BDNF from different developmental nanofiber frameworks over time (Fig. 4).

The initial observations indicated that the release of growth factors was more effectively controlled over the long term in the core-shell model than in other electrospinning models. The release rate profile of the BDNF in the core-shell nanofibrous sample followed a relatively constant and gradual pattern. In contrast, in the bilateral dual mechanism, sharp and clear peaks showed the leaving of BDNF, and burst releases were observed. The final BDNF release rate shows a lower level in layer-by-layer and bilateral dual mechanisms. According to the BDNF release profile, optimum conditions for electrospinning PVA/PCL nanofibers (30/70 v/v) with core-shell mechanisms were selected.

**Fig. 5:**
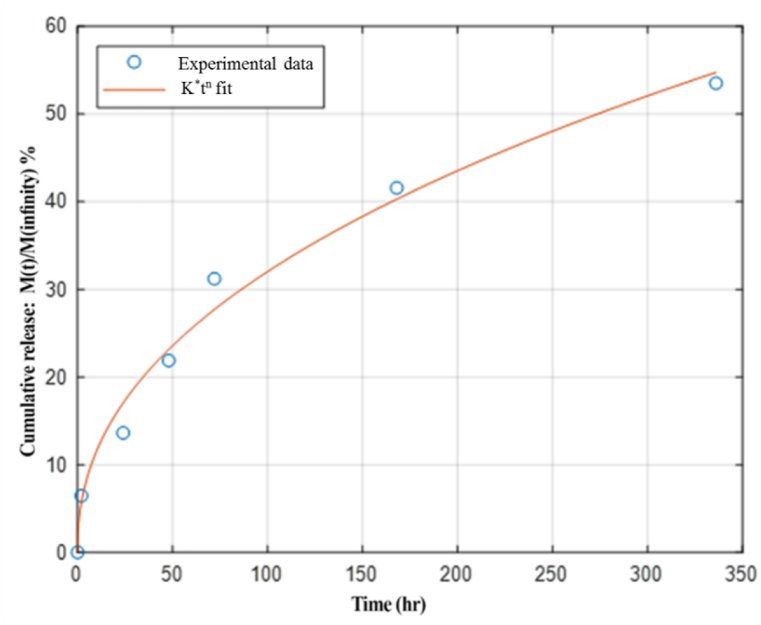
*Invitro* cumulated BDNF release (%) study of core-shell electrospun nanofiber under the optimum conditions in a PBS environment (pH 7.2) over 14 days. K∗t^n^ fit.

The study data and release pattern were evaluated using a Bradford assay on a core-shell electrospun mat. The results indicate a controlled and sustained release of BDNF over 14 days, resulting in a cumulative release of 53.52 ± 0.035 %. The sample demonstrated a relatively rapid drug release rate during the initial 72 h. The release profile of the core-shell structure exhibited a linear sustained release without an initial burst effect. Approximately 31.21 % ± 0.030 of BDNF in the PVA core was gradually released within 72 h (Fig. 5). Subsequently, the release rate accelerated from 72 to 336 h, resulting in 53.52 ± 0.035 % of BDNF being released. The results should adhere to an appropriate mathematical model [26] to accurately predict the release of BDNF from nanofibers *invitro*. The dataset depicted in Fig. 5 aligns closely with the Korsmeyer-Peppas model, demonstrating a strong correlation coefficient of 0.981.

The release of a drug from a polymer matrix can occur through two predominant mechanisms: drug release from the matrix swelling and polymer matrix degradation. In the Korsmeyer-Peppas model, the release exponent “n" is approximately 0.429, which correlates with the diffusion exponent “n" in the Fickian diffusion mechanism, determining the drug release rate. According to the model, PBS can permeate the NGC matrix, dissolve embedded BDNF, and predominantly regulate BDNF release through diffusion, with degradation playing a minor role. The conduit design enables continuous BDNF release, which is achieved via diffusion.

The strategy for releasing growth factors is crucial, and abrupt release should be avoided. The sustained release enhances the efficacy of a drug delivery system. Controlled release of neurotrophic factors promotes axon growth and peripheral nerve regeneration [24,27]. This study shows that combining BDNF with PVA enhances its long-term delivery and effectiveness over 14 days. The inherent properties of PCL allow for less degradation in the shell layers, which helps control the drug's release. Conduits loaded with BDNF effectively deliver drugs during cell differentiation, thus improving the healing process [28,29]. Furthermore, our results indicate that BDNF significantly affects dental pulp stem cells' chemotactic behavior and differentiation. However, it is essential to acknowledge that the temporary elevation of growth factors is necessary to prevent immune responses against regenerating axons [30].

### 3.4 Characteristics ATR-FTIR peaks for PCL, PVA, and two-layer NGC

**Fig. 6:**
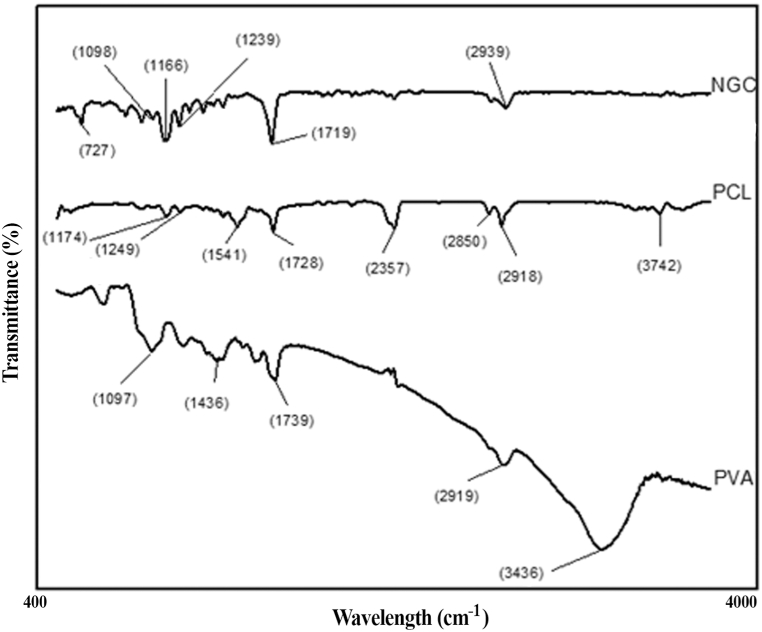
ATR-FTIR spectra of PVA, PCL, and two-layer NGC in the 4000-400 cm-1 range are demonstrated.

analysis confirms the presence of chemical interactions in polymer blends. Shifts or broadening of peaks in the blend's IR spectrum indicate such interactions [31]. Fig. 6 illustrates the PCL/PVA two-layer NGC's ATR-FTIR pattern, confirming solvent evaporation through the analysis.

In the NGC spectra, the peaks at 1098 and 1719 cm^−1^ indicate the PVA phase, while those at 1467 and 1239 cm^−1^ correspond to the C–O group of PVA. Other peaks point to the presence of the PCL phase. The absorption band at 2939 cm^−1^ signifies symmetric CH2 stretching, while the bands at 1719 cm^−1^ indicate C=O stretching. The band at 1166 cm^−1^ represents C–O stretching, and the peak around 727 cm^−1^ corresponds to C–C stretching. Analysis reveals that the intensity of the carbonyl peak at 1719 cm^−1^ and the band centered at 1166 cm^−1^ provides compelling evidence supporting the formation of bonds between the carboxyl and amine groups of PVA.

The characteristic peaks in ATR-FTIR spectra of PCL after electrospinning are given as follows: The peak at 1174.4 cm^−1^ for symmetric COC stretching; 1174 for C–H stretching; 1249 cm^−1^ asymmetric COC stretching and C–C stretching; 1467 cm^−1^ for C–H bending; 1541 cm^−1^ for C=O stretching; 1728 cm^−1^ carbonyl stretching; The two peaks at 2918 cm^−1^ and 2850 cm^−1^ correspond to asymmetric CH_2_ stretching and symmetric CH_2_ stretching, respectively. Additionally, there is a peak at 3742 cm^−1^ for OH stretching [31,32].

The recorded spectra exhibit a broad absorbance at 3436 cm^−1^ for pure PVA, attributable to the O–H and N–H bonds of the PVA hydroxyl and amine groups, respectively. In addition, the presence of the carbonyl group is indicated by bands at 1739 cm^−1^, while the band at 1097 cm^−1^ corresponds to the C–O stretching of acetyl groups present on the PVA backbone. The corresponding bending and wagging of CH_2_ vibrations are observed at 1436 cm^−1^ [33].

### 3.5 Mechanical analysis of the NGC

#### 3.5.1 Tensile test

To qualify as an NGC, a material must meet several mechanical standards, including stress resistance, flexibility, and elasticity [34]. Table 2 indicates that the mechanical properties of 3 types of samples have been analyzed using tensile strength, Young's modulus, and elongation at break.

**Table 2:** Mechanical characteristics of fibrous PCL, PVA, and two-layer conduit. Experiments were done with three replicates.

	Nanofiber mat	3D structure	Two-layer NGC
Ultimate strength (Mpa)	1.25	3.68	2.57
Ultimate strain (%)	3.70	13.06	14.12
Young's modulus (Mpa)	0.4381	0.3973	0.3236
Elongation at break (mm)	0.85	1.50	1.62

**Fig. 7:**
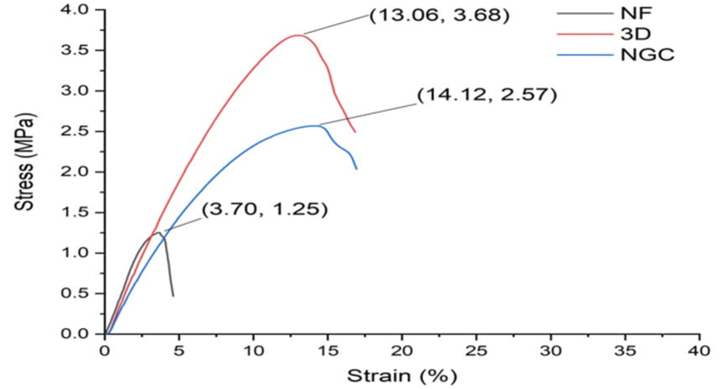
Stress-strain curves of samples: nanofibers mats, 3D layer, and two-layer conduit (Each sample was triplicated to produce the graph).

Nanofibers demonstrated weak strength in tensile tests, so the structure's stability was associated with its 3D arrangement. Adding an external layer enhanced the elasticity of the fiber mat in the internal layer, as shown in Fig. 7. The presence of two layers notably influenced the mechanical properties of the developed NGCs. The structure's plot encompasses a linear elastic region of constant stress. All samples showed Young's modulus values below 0.5 MPa. Compared to the other samples, the 3D sample displayed superior resistance to deformation and ultimate load in terms of ultimate tensile strength.

Furthermore, the nanofiber sample exhibited a significant elastic range and less elastic deformation than the others. The elongation at break is an important indicator of a material's ability to withstand shape changes without fracturing. In the comparison, the 3D sample demonstrated the highest degree of elasticity, as evidenced by its elongation at break measurement. Conversely, the fiber mats exhibited the lowest elasticity.

The observed results generally indicated that the two-layered NGC exhibited higher elongation at break and lesser Young's modulus values compared to 3D and nanofiber samples. Also, the two-layer NGC structure exhibits greater strain than the 3D and fiber mat. On the contrary, the 3D layer and two-layer conduit required more force to break. How the fibers are connected and stacked can affect the structure's structural integrity and mechanical properties. This, in turn, can significantly impact how cells integrate and proliferate [35].

#### 3.5.2 Wettability assessment

**Fig. 8:**
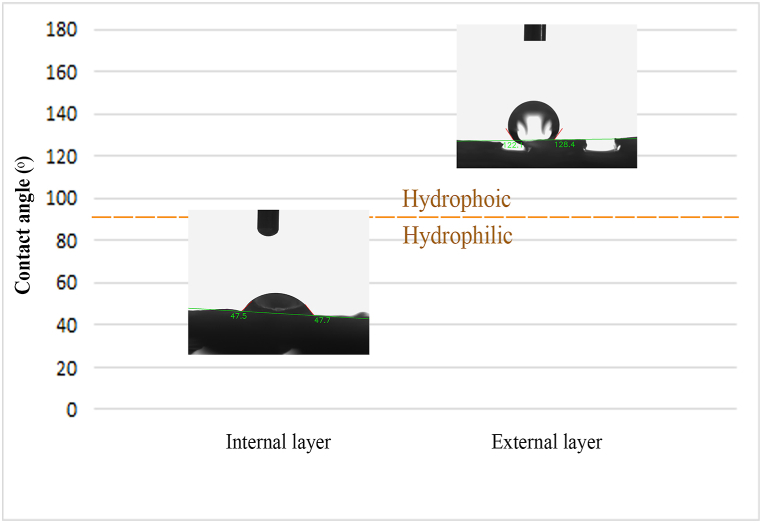
Contact angles and water drop images on the inner and outer layers of the NGC were measured.

The wettability of NGC's surface is critical for cell adhesion, migration, proliferation, and hydrolytic degradation processes [36]. This study combined a hydrophilic polymer, PVA, with a hydrophobic polymer, PCL, to enhance the final product's hydrophilic properties. The hydrophobic PCL was chosen for its long-term stability in the body, while the hydrophilic PVA was selected for its excellent biocompatibility. The wettability of water droplets on electrospun fiber mats and 3D structures was evaluated by measuring the contact angle (Fig. 8).

The data presented in Table 3 showcases the contact angle measurements for both the PCL 3D layer and PCL/PVA mats. Notably, the contact angle values for the PCL layer and PVA/PCL core-shell are recorded as 124.21° and 51.8 ± 3.67°, respectively. These values suggest contrasting superhydrophobic behaviors between the outer and inner layers. The experiment has successfully validated the inner layer's efficacy in influencing cell fate rates.

**Table 3:** Water contact angle data for two-layer NGC.

Sample	Water contact angle
3D layer (external layer)	124.21 ± 2.56
Nanofibrous mat (internal layer)	51.8 ± 3.67

The structure's hydrophilic-hydrophobic properties are crucial in tissue culture, as they significantly impact cell adhesion and migration [37]. Substrates made through electrospinning using synthetic polymers such as PCL show lower cell affinity due to inadequate hydrophilicity and a lack of recognition sites. However, incorporating PVA improves the structure's hydrophilicity, leading to a more favorable cellular response [38].

Due to PVA's hydrophilic nature, a cross-linking process is required. Nevertheless, the potential toxicity of cross-linkers presents considerable challenges [39]. The core-shell structure presents a promising alternative to cross-linking, effectively controlling burst release and broadening the scope of PVA's applications in medicine and tissue engineering. Adequate water absorption is essential for facilitating the functionality of tissue-engineered structures in transporting nutrients and proliferating cells [40]. PCL nanofibers have minimal water absorption due to their hydrophobic characteristics, unlike PVA fibers, which have enhanced absorption because of their hydrophilic properties and hydroxyl groups.

Our working hypothesis suggests that the hydrophilicity of PVA in the core of the fibers creates a hydrogel-like structure that envelops the hydrophobicity of PCL in the shell. Therefore, the core-shell structure derived from coaxial electrospinning enhances cell proliferation.

#### 3.5.3 *Invitro* degradation rate

Water uptake facilitates the hydrolytic degradation process. Hydrolytic degradation involves bulk degradation, slight weight loss, and a decrease in Mw. Measuring weight loss quantifies polymer degradation directly [41].

**Fig. 9:**
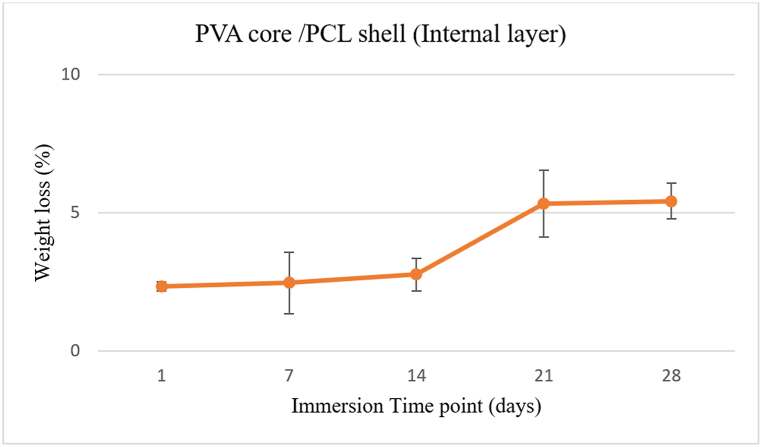
Mass reduction curve of the inner part of the conduit at different incubation time points.

In this study, samples were incubated in pH = 7.2 buffer solution for 28 days in an *invitro* degradation study. The mass loss percentage was calculated for degradation rate determination based on Fig. 9. After 24 h, the weight began to decrease. NGC quickly lost 2.3 % of its initial weight due to PVA dissolving. Only 30 % of NGC is PVA, protected by a PCL shell, preventing rapid degradation. PCL with a hydrophobic structure preserves PVA from quick degradation. Despite being hydrophobic, the PCL degraded slowly, but the fibrous PCL with high porosity allowed water to penetrate the PVA core. PVA fibers were highly hydrophilic and degraded rapidly [42].

Data shows an average weight loss of tested samples over 28 days. The degradation rate increased slowly from day 1 to day 28. Samples on days 14–21 showed a significant mass reduction. The core-shell structure of the NGC inner layer had a low degradation rate of 5.42 ± 0.64 % after 28 days in PBS.

The outcome shows how well the coaxial electrospinning method creates a core-shell structure membrane to overcome the issue of cross-linking in highly degradable membranes.

The core-shell structure based on degradation rate is suitable for surgical use due to its effect on tissue attachment, proliferation, vascularization, and foreign body reaction [43]. Furthermore, PVA rapid degradation aids in BDNF release from the NGC core. The results of the degradation tests conducted *invitro* showed that the hydrophilic PVA component significantly impacts the structure's ability to absorb water and its mass loss due to hydrolysis. The process of polymer hydrolysis in PBS is complex and involves various mechanisms, such as the degradation of side and backbone chains and the breaking of intermolecular cross-link bonds, which depend on the type of polymer [44]. In this research, fiber degradation was observed slowly for PCL single-layered NGCs (data not shown).

Biomaterials with increased water absorption exhibit accelerated degradation, suggesting that PVA structures may degrade more rapidly due to their elevated swelling capacity [35]. Additionally, the PVA's water absorption resulted in swelling, enlargement of fiber diameter, and a decrease in pore size.

The material degradation characteristics significantly affect scaffolds' mechanical properties, so an optimal degradation rate is needed to ensure sufficient mechanical support during neo-tissue development [45,46]. Since PCL is the main component of the conduit's structure, the results are more closely related to PCL than PVA. The core of PVA has minimal structural effects. Therefore, the fibrous core-shell architecture maintains its structural integrity and reduces tissue attachment and foreign body reactions [43]. The stability of PCL ensures support for the conduit until nerve tissue repair is complete. These results demonstrate the importance of the core-shell structure in tissue engineering.

#### 3.5.4 Cytotoxicity study of the NGC

**Fig. 10:**
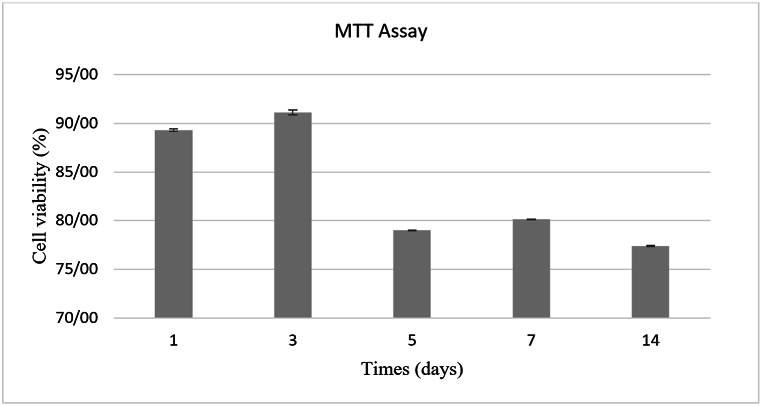
MTT tested cell viability in contact with leachates of the prepared NGC.

An assessment was conducted to evaluate the cytotoxicity of NGC using cell lines and the indirect MTT assay. The data analysis involved using optical density values to determine the cell viability percentage and evaluate biocompatibility. Fig. 10 presents bar graphs showing the cell viability of NGC for different incubation periods (1–14 days). The NGC demonstrated enhanced cell proliferation after 72 h of culture, with a slight increase in viability from 89.34 % to 91.14 %. The proliferative properties may indicate the effects of BDNF release. Based on the ISO 10993–5 protocol, cytotoxicity is determined by reducing cell viability to less than 70 % of the negative control [47], so the result of the cytotoxicity test indicates that cell viability remained consistently above 77 % throughout the study period, with no detectable release of cytotoxic substances from the two-layer conduits.

Furthermore, no discernible toxicity was observed over 14 days under typical usage conditions, suggesting that the NGC supports cell adhesion, proliferation, and distribution. However, excessive fibroblast proliferation during healing can lead to fibrosis. Therefore, the conduit must not induce excessive fibroblast activity or scar tissue formation to enhance wound healing [48].

### 3.6 Cell behavior in NGC

#### 3.6.1 *Invitro* cell-conduit interaction

**Fig. 11:**
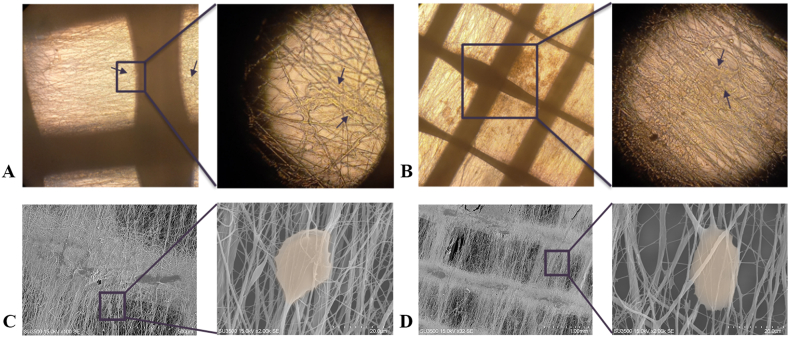
Optical microscope images of attached hDPSCs cultured on the conduit after 2 (A) and 14 (B) days. SEM images of attached hDPSCs cultured in the conduit after 2 (C) and 14 (D) days.

The morphology, adhesion, and proliferation rate of hDPSCs on the internal layer of the conduit were observed using an inverted optical microscope (Fig. 11A and B) and SEM (Fig. 11C and D). Fig. 11 presents the cell growth results after 2 and 14 days. The SEM images showed that the hDPSCs cultured on nanofibers adapted well to the surface, with the growth indicated by the arrows. PCL and PVA are synthetic polymers unsuitable for cell attachment, but the micro- and nanofibrous structure of the inner layer promotes cell adhesion and proliferation [49].

### 3.7 Characterization of differentiated cells on NGCs

#### 3.7.1 SC-specific gene expression

**Fig. 12:**
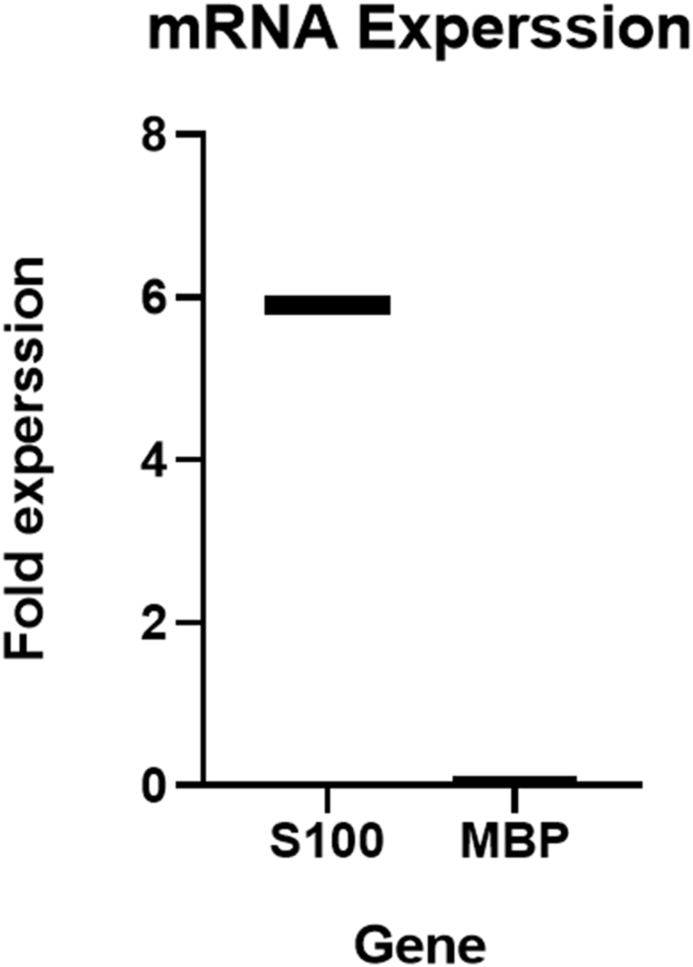
Quantitative expression status of the S100 and MBP genes specific to Schwann cells in cultured hDPSCs on the NGC (differentiated stem cells) after two weeks. Primary stem cells were used as positive controls.

Quantitative analysis was performed using PCR to confirm the presence of specific Schwann cell markers, including S100 and MBP, relative to the reference gene GAPDH. The mRNA levels of SC-specific markers were calculated in total RNAs from undifferentiated SCs and differentiated SCs (Fig. 12). The quantitative analysis confirmed that after 14 days of induction, the cultivated hDPSCs on the conduit had differentiated into SCs. As can be seen, the expression of S100 in differentiated hDPSCs on the conduit showed a 5.89-fold increase compared to undifferentiated hDPSCs.

### 3.8 Immunofluorescence

#### 3.8.1 hDPSCs differentiate into cells expressing Schwann cell markers

Immunofluorescence was utilized to assess the efficiency and temporal progression of Schwann cell markers expression in differentiated mesenchymal stem cells (hDPSCs). The investigation centered on the molecular-level expression of S100 and MBP proteins, common markers in neuronal development and regeneration. The Immunofluorescence staining for S100 and MBP markers exhibited significant immunoreactivity in cultured mesenchymal stem cells within the conduit after 14 days. Conversely, undifferentiated Schwann cells demonstrated non-immunoreactivity, although accompanying images were not included.

**Fig. 13:**
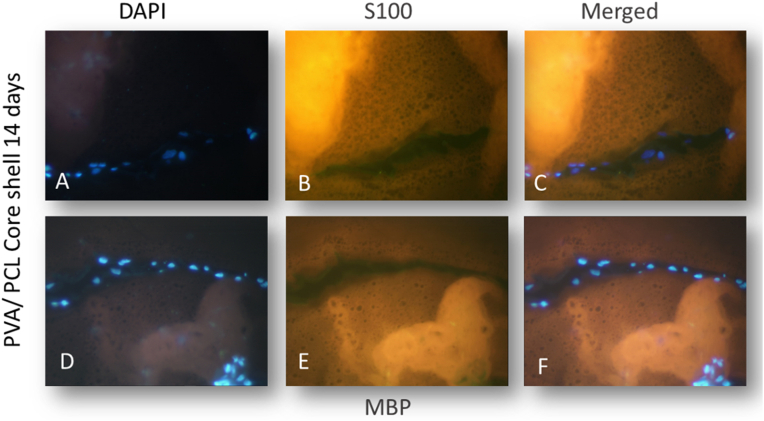
Cultivated hDPSCs in the conduit were characterized using anti-S100 and anti-MBP antibodies after two weeks. The findings were observed through fluorescence microscopy of nuclei staining (A and D), IF staining (B and E) of some differentiated hDPSCs in the conduit, and the merged images (C and F).

In a specific region within the conduit, there is a difference in the number of visible cells after nuclei staining with DAPI and SC-specific antibodies. The number of cells labeled with DAPI is higher than those labeled with antibodies, indicating the presence of undifferentiated stem cells and the heterogeneous differentiation of hDPSCs into Schwann-like cells (Fig. 13).

The results of real-time and immunofluorescent assessments show that our innovative approach can generate a two-layer neural conduit with biomimetic and topographic features, facilitating longitudinal and chemotactic cell growth *invitro*. Based on our findings, it is suggested that two-layer conduits have the potential to enhance the longitudinal development of stem cells and facilitate their differentiation into Schwann-like cells.

## 4 Conclusion

The process of mimicking neural tissues *invitro* comes with significant technical challenges. One major obstacle is the selection of biomaterials. Bio-printable materials comprise only a small portion of the biomaterials used in neural tissue regeneration, and achieving a balance between cell support and printability remains arduous. In our research, we opted for two FDA-approved biomaterials, recognizing that the neural conduit must possess sufficient durability to maintain the regenerative microenvironment over time. The hollow nerve conduits were fabricated through 3D printing, employing PCL and a slowly biodegradable nanofiber for the intraluminal (PVA/PCL core-shell). We used a two-layered framework to amplify the mechanical reinforcement and reduce the risk of tissue collapse post-grafting.

Since the morphogenic gradient of signaling molecules plays an important role in organ development and regeneration, regulating biochemical factors can significantly enhance the laboratory model's efficacy. The sustained release of BDNF is crucial for the regeneration of peripheral nerves. However, its therapeutic effectiveness is limited due to its short half-life and rapid degradation *in vivo*. The core-shell electrospinning technique was utilized to improve the sustained release of growth factors and to create topographical conditions for cellular proliferation and differentiation. Fibrous substrates within the lumen provide the necessary space for tissue regeneration. Incorporating growth factors within conduit walls has the potential to augment axonal growth more effectively than luminal fillings. Fabricating such conduits could expedite their utilization as three-dimensional models or organoids, thereby facilitating laboratory investigations into neural tissue responses to pharmacological agents.

## CRediT authorship contribution statement

**Massoumeh Jabbari Fakhr:** Investigation, Writing – original draft. **Mohsen Eslami Farsani:** Writing – review & editing. **Leyla Fath-Bayati:** Writing – review & editing. **Reihaneh Seyedebrahimi:** Writing – review & editing. **Mehdi Sadrjahani:** Validation. **Fatemeh Kavakebian:** Investigation. **Alireza Rezapour:** Funding acquisition, Project administration, Supervision, Writing – review & editing.

## Data availability

The datasets generated during and/or analyzed during the current study are available from the corresponding author upon reasonable request.

## Declarations

Ethics approval and consent to participate: Not applicable.

## Consent for publication

Not applicable.

## Funding

This work was supported by the Qom University of Medical Sciences (MUQ) through research Grant No. IR.MUQ.REC.1400.180.

## Declaration of competing interest

The authors declare that they have no known competing financial interests or personal relationships that could have appeared to influence the work reported in this paper.
